# A novel p–n junction UiO-66/AgInS_2_ for photodegradation of enrofloxacin under visible light

**DOI:** 10.55730/1300-0527.3747

**Published:** 2025-02-25

**Authors:** Sajedeh Sadat HASHEMIPOOR, Reza FAZAELI, Hamid Reza MOGHADAM ZADEH, Mehdi ARJOMAND

**Affiliations:** Department of Chemical Engineering, Faculty of Technical & Engineering, South Tehran Branch, Islamic Azad University, Tehran, Iran

**Keywords:** UiO-66, AgInS_2_, photocatalysis, degradation, enrofloxacin, Box-Behnken design

## Abstract

The formation of a heterogeneous junction between photocatalysts may be a potential solution for removing medicinal compounds from wastewater, which is crucial due to the environmental and health risks. The present study aims to investigate the efficacy of UiO-66/AgInS_2_ p–n junction photocatalyst—a Zr-based metal-organic framework—in degradation of enrofloxacin (ENR) antibiotic using visible light. UiO-66, AgInS_2_ (AIS), and UiO-66/AIS semiconductors were characterized using various techniques. The comparative analysis of particle structural parameters utilized diverse methodologies, and response surface methodology (RSM) was applied for the optimization of ENR degradation. The degradation kinetics of ENR were investigated. We identified UiO-66/AIS as a p–n junction semiconductor with a 1.71 eV direct band gap. Mott–Schottky analysis yielded E_CB_ and E_VB_ values of 1.72 V and 0.64 V for UiO-66 and −0.5 V and 1.05 V for AIS, respectively. The size-strain plot (SSP) method was determined to be the most effective in determining crystal dimensions, and a UiO-66/AIS p–n junction was instrumental in achieving 94.32% ENR degradation in a very short time using a pseudo-second-order kinetic model. The findings of this empirical study indicate that the UiO-66/AIS p–n junction has demonstrated exceptional efficiency in the removal of the ENR drug.

## 1. Introduction

Antibiotics are widely employed in the management of microbial infections affecting both humans and animals. However, their use is associated with several undesirable outcomes, such as antibacterial resistance, genotoxicity, and gastrointestinal disturbances [1–4]. Enrofloxacin (ENR), a veterinary antibiotic marketed under the trade name Baytril, is widely used to treat respiratory infections and salmonellosis in ruminants. ENR has strong antibacterial efficacy against gram-negative bacteria, certain gram-positive bacteria, and mycoplasmas. Nonetheless, the release of this antibiotic into the environment without proper treatment can have a detrimental impact on humans and ecosystems. Therefore, it is imperative to remove or diminish the concentration of ENR before releasing it into the environment [5–7]. Researchers have employed a variety of techniques for wastewater treatment, such as membrane [8], adsorption [9], reverse osmosis [10], and photocatalytic methods [11,12]. Photocatalysis is a novel technology that offers numerous benefits in this regard. In this approach, semiconductors use energy from a radiation source to generate free radicals that decompose pollutants. This process has been shown to be effective in several studies [13–15].

Metal-organic frameworks (MOFs) have garnered significant scholarly attention in recent decades, attributed to their nanoporous characteristics. These frameworks are composed of inorganic nodes interconnected by organic linkers [16]. The synthesis of MOFs involves the combination of various metal ions and organic linkers in an appropriate solvent, leading to the formation of intricate three-dimensional crystal structures that exhibit unique properties with applications in diverse fields such as gas storage, catalysis, photocatalysis, and drug delivery [17–21]. MOFs are essentially an array of metal centers where ligands bridge the gaps. As each metal center is connected to more than one ligand, an infinite arrangement of metal centers is created. The set of physical and chemical properties of inorganic and organic components, as well as the cooperation between these two components in the polymer, give MOFs attractive and unique features. More generally, coordination polymers, which include MOFs, have become increasingly popular and are highly sought-after for their microporous nature and diverse range of applications [22]. UiO-66 has been identified as a viable option for producing a photocatalyst base among MOFs due to the fact that the holes present in this composition are relatively small in size compared to other particles. It is a well-established fact that the smaller the size of the filler particles, the smaller the chances of nonselective hole formation at the polymer-filler interface. Additionally, UiO-66 exhibits a remarkably high specific surface area, which in turn increases the adsorption process. This makes UiO-66 an ideal candidate for the development of a photocatalyst base [23,24].

AgInS_2_ (AIS) is a significant ternary chalcogenide utilized in the semiconductors industry. It possesses a minor band gap ranging from 1.87 to 2.03 eV and exhibits excellent visible region absorption capabilities [25]. The orthorhombic AIS, tetrahedral InS_4_, and AgS_4_ combine to form a wurtzite-like structure within the unit cell, as observed in previous studies [26]. Photocatalytic activity can be improved by the formation of a heterogeneous junction between UiO-66 and AIS. Photocatalysts with heterogeneous n and p junctions increases the lifetime of produced electrons and holes and enable faster electron and hole transfer. Multimembered compounds can exist in various forms. One member of the photocatalyst can be loaded onto another as a substrate, or two members can be combined in the same or different ratios. This results in an increase in light absorption range and promotes efficient photocatalysis [27–29].

Response surface methodology (RSM) constitutes a collection of mathematical and statistical techniques that are exceptionally beneficial for the modeling and examination of intricate issues wherein the response variable is influenced by multiple independent variables [30]. The main goal of RSM is to enhance and fine-tune the response variables through a systematic and efficient approach that involves experimentation and data analysis [31,32]. By utilizing RSM, researchers can gain valuable insights into the relationships between the independent variables and the response variable, leading to optimal solutions and improved outcomes. During the process, the RSM serves as a valuable tool for describing quality indicators. In most practical experiments, several factors contribute to the quality and performance of a product, and it becomes imperative to investigate these factors. In the context of response surface optimization, the independent variables are defined as input variables, and their impact on the dependent output variables is studied. The most significant advantage of RSM lies in its ability to reduce the number of experiments necessary to evaluate multiple parameters and their interrelationships, as evidenced by prior research [33,34].

As previously stated, the discharge of wastewater laden with medicinal compounds such as ENR poses a significant threat to the environment and, consequently, to human health. Therefore, developing an effective method to eliminate these chemical substances from aquatic environments has become imperative. As such, there is an urgent requirement for an efficacious system to purify water resources.

Huijie et al. demonstrated that the synergistic interplay between the AgIn_5_S_8_/Zn_4_In_2_S_7_ heterojunction and ultrathin GO effectively enhanced the separation and transfer of photogenerated carriers, resulting in a 96% degradation rate of tetracycline (TC) [35]. Anhu et al. developed highly effective and stable NiIn_2_S_4_/UiO-66 photocatalysts, demonstrating nearly complete degradation of 20 mg/L TC under visible light in 1 h at a NiIn_2_S_4_ to UiO-66 mass ratio of 0.5:1 and pH 11, with a sustained degradation rate of 90% over six cycles [36]. Quach et al. investigated the photocatalytic efficacy of samarium orthovanadate (SmVO_4_) nanorods combined with UiO-66-NH_2_ for the degradation of TC antibiotics under simulated solar irradiation, revealing that the SmVO_4_/UiO-66-NH_2_ nanocomposites establish a direct Z-scheme heterojunction facilitating a 50% degradation efficiency in 90 min, in contrast to the 30% degradation observed with bare-SmVO_4_ nanorods [37].

The present study aims to optimize the removal of ENR antibiotics using UiO-66/AIS photocatalyst in the presence of visible light in aqueous solutions based on RSM. Furthermore, the dimensions of the crystals corresponding to UiO-66, AIS, and UiO-66/AIS particles were examined through different methodologies including Debye–Scherrer (DS), Williamson–Hall (WH), and size-strain plot (SSP). The findings of this research have significant implications for the development of advanced systems to treat wastewater and mitigate the impact of medicinal compounds on the environment and human health.

## 2. Materials and methods

### 2.1. Chemicals

Zirconium(IV) chloride (Zr(Cl_4_)) with a purity of ≥99.9%, terephthalic acid (C_8_H_6_O_4_) with a purity of 98%, N,N-dimethylformamide anhydrous (DMF) with a purity of 99.8% (C_3_H_7_NO), silver nitrate (AgNO_3_) with a purity of ≥99.0%, indium(III) nitrate hydrate (In(NO_3_)_3_) with a purity of 99.9%, tannic acid (TA) with a purity of 99.9% (C_76_H_52_O_46_), sodium hydroxide (NaOH) with a purity of ≥99%, ethanol (C_2_H_6_O) with a purity of 98%, and ENR (C_19_H_22_FN_3_O_3_) with a purity of ≥99% were obtained from Sigma Aldrich Co.

### 2.2. Synthesis of UiO-66

In this experiment, a mixture of 0.52 g of Zr(Cl_4_) and 0.37 g of C_8_H_6_O_4_ was added separately to 30 mL of DMF. The resulting mixture was stirred for 20 min at 250 rpm until it was well-mixed. Subsequently, the dissolved solutions were placed in a 100 mL Teflon autoclave, which was sealed and heated at 120 °C for 24 h. After the autoclave was removed from the oven, the resulting precipitate was washed multiple times with DMF. The resultant white sediment was subjected to a drying process in an oven.

### 2.3. Synthesis of AIS

AgNO_3_, In(NO_3_)_3_, and C_76_H_52_O_46_ were individually added to 50 mL of distilled water in a 1:1:2 molar ratio, agitated for 15 min, and then combined to produce a transparent solution. The pH of the solution was modified to 10 by introducing 1 M NaOH. The resulting mixture was then transferred to an autoclave and held at 180 °C for 24 h. The final product was centrifuged, washed several times with deionized water, and subsequently dried at 80 °C for 6 h.

### 2.4. Synthesis of the UiO-66/AIS p–n junction

The UiO-66/AIS p–n junction was prepared by incorporating weight percentages of 5%, 10%, 15%, and 20% AIS into UiO-66. The varying amounts of AIS were added to UiO-66 in 100 mL of C_2_H_6_O and stirred on a magnetic stirrer for 8 h. The ensuing mixtures were subjected to centrifugation, and the resulting products were dried in an oven at 400 °C for 2 h.

### 2.5. ENR adsorption and photocatalytic degradation process

UiO-66/AIS p–n junction samples containing variable percentages of AIS were utilized for ENR photocatalytic degradation experiments. A batch reactor with a visible lamp was used to mix the reaction solutions. The reactor was constructed from wood and measured 45 × 45 × 50 cm in length, width, and height. Visible lamps were mounted on all four sides of the reactor. To prevent visible light scattering, the internal region of the reactor was fully coated with aluminum sheeting. A magnetic stirrer was housed inside the reactor to facilitate the mixing process. The experimental procedure involved the addition of a specific quantity of photocatalyst and ENR, totaling 150 mL, to a pollutant solution, which was then subjected to visible light irradiation using 4 lamps of 25 W power and 400–800 nm continuous wavelength. Similarly, in order to investigate the adsorption of the synthesized photocatalysts, experiments were conducted in the absence of visible light, i.e. in the dark. Stirring the solution at a predetermined rate with the magnetic stirrer was necessary to ensure complete dispersion of the photocatalyst. Centrifugation was conducted for a minute at 70,000 rpm after each sample had been irradiated for the designated time period in order to separate the photocatalyst from the solution and measure the ENR concentration. The pollutant concentration was assessed using a spectrophotometer. The device’s wavelength was set to 280 nm, the maximum wavelength of ENR absorption, in order to determine the concentration. The equation to compute the percentage of pollutant degradation in the photocatalyst process was as follows:


(1) $$\text{Degradation}(\%)=\frac{c_{\text{o}}-c_{e}}{c_{\text{o}}}\times 100$$

The initial concentration of the ENR, denoted as C_o_, and the final concentration of the ENR after undergoing photocatalytic degradation, denoted as C_e_, are important factors to consider in the study of pollutant degradation.

### 2.6. Effect of photocatalyst type

The influence of UiO-66/AIS containing varying weight proportions (0, 5, 10, 15, and 20%) on the decomposition of ENR medication was investigated. The study entailed subjecting 50 mL of ENR solution with a concentration of 100 mg/L at pH 7 to irradiation for a period of 5–15 min in the presence of 0.01 grams of photocatalyst. Also, in order to investigate the adsorption of the synthesized photocatalysts, experiments were carried out under the mentioned conditions in the dark and in the absence of visible light.

### 2.7. Design of experiment

Among the various RSMs, the Box–Behnken design (BBD) stands out due to its minimal number of tests required, comprising only 27 experiments for 4 variables. The RSM model used in the current study incorporates a complete quadratic model equation, which can be expressed in the following manner:


(2) $$Y=\beta_{0}+\sum_{j=1}^{k}\beta_{j}X_{j}+\sum_{j=1}^{k}\beta_{jj}X_{j}^{2}+\sum_{i}\sum_{<j=2}^{k}\beta_{ij}X_{i}X_{j}+e_{i}$$

The ensuing model for the response variable (Y) is structured in a polynomial equation of autonomous variables, with a design that is experimental in nature. The equation for the aforementioned response (Y) involves x_i_ and x_j_ as the independent variables, while b_0_ is a fixed value, and b_i_, b_ii_, and b_ij_ are associated with the coefficients of linear regression, quadratic, and interaction, respectively. During the process of variance analysis, the statistical significance of the quadratic models is determined by means of F value, and the significance level (p-value) is set at 0.05. If the calculated F-value exceeds the stipulated F-value, then the p-value will be much lower. This underlines the relevance of the statistical model.

At this stage, an assessment was performed regarding the influence of various factors, including pH, mass of the photocatalyst, concentration of ENR, and duration of exposure to visible light, on the effectiveness of the degradation mechanism. The photodegradation of antibiotics was carried out using 4 lamps with a 25 W capacity. The pH of the solution was adjusted to three levels (4, 7, and 10) using NaOH and HNO_3_. Different amounts of UiO-66/AIS (15%) (0.01, 0.055, 0.1 g) were introduced to the solution. The desired concentrations of ENR (100, 200, 300 mg/L) were prepared by diluting a certain amount of ENR stock solution in a volume of 50 mL for experimentation purposes. Sampling was performed at 5, 10, and 15 min intervals from the irradiated suspension (Table 1).

### 2.8. Reaction kinetics

To explore the degradation kinetics of ENR, a systematic study was conducted wherein 150 mL of a pharmaceutical solution with a concentration of 108 mg/L was subjected to visible light exposure for varying durations spanning from 1 to 12 min, in conjunction with 0.015 g of UiO-66/AIS (15%). The results obtained from this study were assessed using the pseudo-first- and pseudo-second-order kinetic models, as well as the equations related to intraparticle diffusion control [38–40].

## 3. Results and discussions

### 3.1. Characterization

#### 3.1.1. X-ray diffraction (XRD)

**Figure 1:**
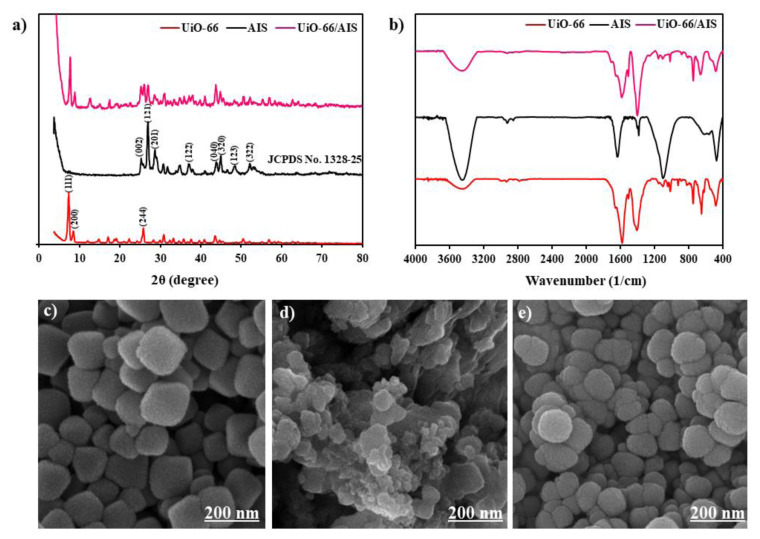
a) X-ray diffraction pattern for UiO-66, AIS, and UiO-66/AIS, b) FT-IR peaks for UiO-66, AIS, and UiO-66/AIS, SEM images for c) UiO-66, d) AIS, e) UiO-66/AIS particles.

Figure 1a displays an XRD curve. It is noteworthy that the presence of peaks exhibiting a narrow distribution and high intensity in the XRD curve is indicative of high crystallinity of the particles. Furthermore, the XRD curve exhibits characteristic and strong peaks at angles of 7.5°, 8.4°, and 25.8°, which are reflected from corresponding crystal planes (111), (200), and (244), respectively. Generally, these peaks are consistent with the XRD patterns previously reported for UiO-66. The weak peak where 2θ equals 25° signifies the successful synthesis of UiO-66, confirming the presence of diagonal linkers in the strong interaction with the inorganic component and the lack of structural impurities. Based on the findings obtained from the XRD technique for AIS, the observed peaks are in agreement with the standard structure of AIS (JCPDS No. 1328–25). The dominant peaks located at 2θ of 26.6°, 28.4°, 28.8°, 36.8°, 43.7°, 44.8°, 48.0°, and 52.6°, which correspond to (002), (121), (201), (122), (040), (320), (123), and (322), respectively are of particular interest. These peaks are linked to the orthorhombic AIS structure. These findings hold importance as they offer a deeper understanding of the structural characteristics of AIS. The diffractogram of the UiO-66/AIS yields peaks at precise angles of 7.5°, 8.4°, and 25.8°, which can be attributed to UiO-66. In addition, the aforementioned p–n junction exhibits peaks at angles of 26.6°, 28.4°, 28.8°, 36.8°, 44.5°, 48.0°, and 52.6°, which correspond to AIS. This outcome strongly suggests that the p–n junction has been synthesized accurately. In the present study, D-S, W-H, and SSP methodologies were used to determine the crystallite size calculations for UiO-66, AIS, and UiO-66/AIS particles [41,42]. The results of crystal size calculation with different methods are presented in Table 2.

#### 3.1.2. Fourier transform infrared (FT-IR)

Figure 1b displays the examination of specimens with FT-IR spectroscopy. In the UiO-66 spectrum, an intense and wide band at 3444.70 cm^−1^ is observed, related to the presence of an OH group caused by intercrystalline water and condensed water that is physically absorbed inside the cavities. The band observed at 2033.95 cm^−1^ is related to H-stretching bonds. The band at 1578.44 cm^−1^ can be assigned to O–C–O asymmetric stretching in the UiO-66 structure. Bands around 1406.17 and 1505.26 cm^−1^ represent the usual vibration in the C=C and C–C bonds of benzene terephthalic acid rings in the MOF structure, respectively. The bands at about 1044 and 1016 cm^−1^ show the stretching vibration of the Zr–O bond of UiO-66. The spectral features observed at 923.95 and 747.90 cm^−1^ can be attributed to the vibrational modes associated with the CH and OH functional groups within the terephthalic acid ligand. At reduced frequency domains, the bending vibrational modes of CH and OH exhibit a confluence with the principal bands associated with Zr–O modes at 620 and 483.17 cm^−1^. Therefore, the infrared spectrum shows that UiO-66 has been successfully synthesized. In Figure 1b, AIS exhibits symmetric and asymmetric stretching vibrations of the –CH_2_ group at the wavenumber of 2924.36 cm^−1^. The peaks at 1637.80, 1384.56, 1100.54 cm^−1^ show C–S, COOH, C–N stretching vibration bands, respectively. In addition, the plot does not show C–H and S–H stretching vibrational bands at 2552 and 638 cm^−1^, respectively. This means that S–H and C–H bonds are broken and the S atom forms AIS with Ag and In. In UiO-66/AIS the peaks at 480.77, 746.60, and 1154.23 cm^−1^ are related to the Zr–O bond of the MOF. The peaks at 1400.62 and 1507.15 cm^−1^ correspond to O–C–O and C=C in UiO-66. The peak at 1584.50 cm^−1^ is related to C–S at AIS. Moreover, the peak at 3453.97 cm^−1^ is related to an OH group. Therefore, the structure of UiO-66/AIS has been successfully synthesized.

#### 3.1.3. Scanning electron microscope (SEM)

The SEM images used to determine the surface morphology of UiO-66 nanoparticles at different magnifications from 200 nm are shown in Figures 1c–1e. In the images, there are a large number of particles that are placed side by side and the boundary of most particles is clear. The SEM images provide evidence that UiO-66 is made of cubic single crystals with octagonal morphology and clear boundaries that have grown side by side. UiO-66 particles are mostly in the form of cubic crystals. The size of a number of particles varies from 39 to 57 nm. In addition, the porosity of UiO-66 particles is observed, especially at 200 nm magnification. Based on the results, the AIS particles are completely uniform and have nano dimensions. Moreover, the particles of UiO-66/AIS (15%) are shown in Figure 1e. It can be concluded that the particles of AIS are well loaded on UiO-66.

#### 3.1.4. Energy dispersive X-ray spectroscopy (EDS)

**Figure 2:**
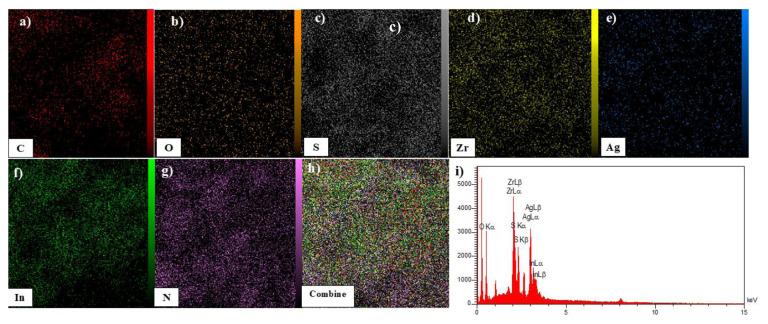
EDS Map images of a) C (red), b) O (orange), c) S (gray), d) Zr (yellow), e) Ag (blue), f) In (green), g) N (purple), h) composite of UiO-66/AIS, and i) EDS analysis.

EDS mapping analysis was conducted to examine the structural characteristics and identify the predominant elements within UiO-66/AIS. The results of this analysis are shown in Figure 2. For this sample, the elements C, O, S, Zr, Ag, In, and N were detected as indicator elements. The weight percentages of these elements were 31.28%, 25.73%, 8.53%, 13.62%, 12.39%, 4.28%, and 4.17%, respectively, confirming the formation process of the UiO-66-AIS structure.

**Figure 3:**
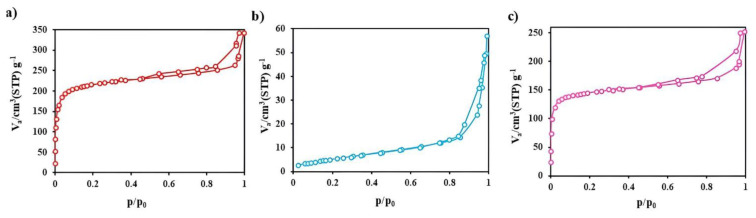
BET-BJH photocatalysts a) UiO-66, b) AIS, c) UiO-66/AIS.

Brunauer–Emmett–Teller (BET) and Barrett–Joyner–Halenda (BJH): The nitrogen adsorption-desorption isotherm for the synthesized UiO-66, as depicted in Figure 3, aligns with type I as categorized by the IUPAC classification. Table 3 delineates the specific surface area of the total void volume and the average diameter of voids formed in UiO-66, with the specific surface area ascertained via the BET methodology. As illustrated in Table 3, the BET specific surface area, the total pore volume, and the average pore diameter for UiO-66 are quantitatively documented as 792.79 m^2^ g^−1^, 0.509 cm^3^ g^−1^, and 2.605 nm, respectively. The substantial surface area and pore volume indicate that UiO-66 materials possess significant adsorption capabilities. The distribution of the pore size radii was evaluated utilizing data derived from the adsorption isotherm in conjunction with the BJH method. The dispersion graph illustrating the pore size distribution of UiO-66 is presented in Figure 3. According to the shape of the distribution, the radius of most of the holes is less than 10 nm, which shows that most of the particles are microporous or mesoporous. However, there are particles with a larger hole radius in the specified range. Furthermore, measurements were taken to determine the specific surface area, total pore volume, and average pore diameter of both AIS and UiO-66/AIS. The results indicated values of 59.21 and 543.14 m^2^ g^−1^, 0.264 and 0.376 cm^3^g^−1^, and 1.240 and 2.776 nm, respectively. These findings suggest that the presence of AIS led to a decrease in specific surface area and total pore volume, while causing an increase in the average diameter of the pores. It can be concluded that AIS is well loaded on UiO-66.

**Figure 4:**
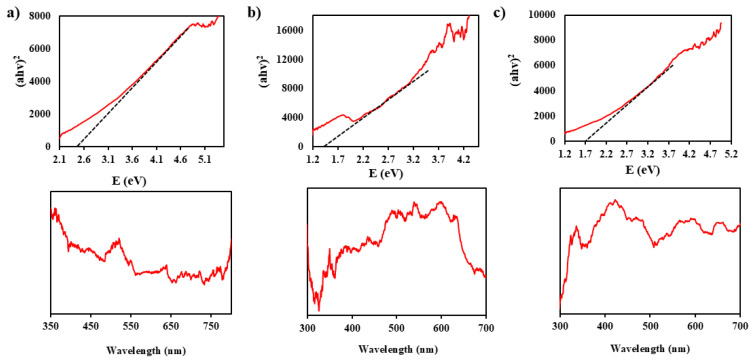
DRS and UV-Vis for catalysts a) UiO-66, b) AIS and c) UiO-66/AIS (15%).

Diffuse reflectance spectroscopy (DRS): DRS was used to measure the energy gap and absorption wavelength of the photocatalysts. The UV-Vis absorption spectrum of the produced photocatalyst in the optical absorption wavelength range of 200–850 nm is shown in Figure 4.

A Tauc plot was used to compute the band gap from the reflection spectra. The associated equation is presented below:


(3) $${\left(\alpha h\nu \right)}^{1/n}=A(h\nu -E_{g})$$

The symbol h denotes the Planck constant, while the symbol ν denotes the frequency in question. Furthermore, α functions as a metric for the absorption coefficient, E_g_ is associated with the energy band gap, and A is identified as a constant of proportionality. Consequently, the calculated band gap energies for UiO-66, AIS, and the UiO-66/AIS p–n junction were 2.36, 1.55, and 1.71 eV, respectively. The UV-visible absorption spectra of the photocatalysts can be seen in Figure 4. Based on the observations, AIS and UiO-66/AIS p–n junction photocatalysts have significant absorption in the visible light range (400–800 nm), indicating that they will have high performance potential under visible light irradiation.

**Figure 5:**
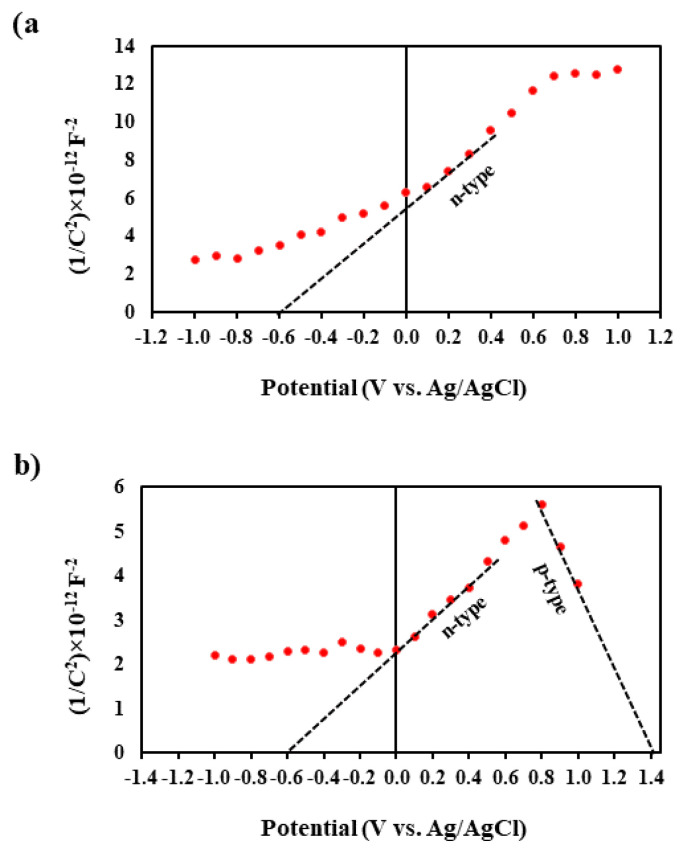
Mott-Schottky plot of a) AIS and b) UiO-66/AIS.

Mott-Schottky: In the context of the UiO-66/AIS p–n junction, electrons and holes show a preference for penetrating the p and n semiconductors, respectively, when p and n semiconductors are linked together. The movement of an electron from the n-type region to the p-type region creates a positively charged donor ion in the n-type region, whereas the migration of a hole results in the formation of a negatively charged acceptor ion in the p-type region. Upon entering the p region, the electron engages in recombination with a hole located on the p side. This process of electron-hole recombination plays a crucial role in the operation and functionality of semiconductor devices. This process is also true for the holes penetrated into the n region. The result is a charged ion and an electric field near the border of n and p semiconductors—an area called the double layer. These ions have a positive and negative charge on the n and p side of the semiconductor, respectively. Due to their very high mass compared to the electron and the hole, they cannot move and neutralize each other, and the electric field prevents the penetration of electrons and holes to the opposite side. The orientation of the electric field is established to be moving from the n region, characterized by an excess of electrons, towards the p region, characterized by a deficiency in electrons due to the presence of electron holes. If the intensity of the electric field reaches such a level that it stops the penetration of electrons and holes, a state of equilibrium is established and the voltage generated at both ends of this region is called a junction voltage. The Mott-Schottky equation was used to examine semiconductor behavior and material flat band potential through the Mott-Schottky curve. The AIS Mott Schottky curve displays a positive slope, signifying an n-type semiconductor. The UiO-66/AIS Mott-Schottky curve has an initially positive slope followed by a negative slope, indicating a p–n type semiconductor (Figure 5). This suggests the coexistence of n- and p-type characteristics within UiO-66/AIS. This phenomenon can be attributed to the successful generation of n- and p-type domains in UiO-66/AIS. The flat band potential of AIS was quantified at −0.6 V in comparison to Ag/AgCl, establishing a reference point for further analysis. Through the application of Equation (4), the adjustment of E_fb_ of AIS concerning Ag/AgCl resulted in a value of −0.4V when referenced against NHE, providing additional insight into the material’s behavior.


(4) $$E_{NHE}=E_{\text{Ag}/\text{AgCl}}+0.197\;\text{V }(\text{pH}=7)$$

In this study, the E_CB_ of the semiconductor in AIS is approximately −0.5V. The value of E_VB_ is determined to be 1.05 V using the relevant equation.


(5) $$E_{g}=E_{VB}-E_{CB}$$

The conduction and valence band potential are denoted by E_CB_ and E_VB_, respectively. The flat band potential of UiO-66 and AIS were +1.43 and −0.6 V (vs. Ag/AgCl), respectively. Through the application of Equation (4) [43], the E_fb_ of UiO-66 and AIS, relative to Ag/AgCl, can be converted to +1.62 and −0.4V (vs. NHE), respectively. Consequently, the E_CB_ values for UiO-66 and AIS were calculated to be +1.72 V and −0.5 V, respectively. Furthermore, equation (18) was utilized to obtain the E_VB_ values of UiO-66 and AIS, which were 0.64 and 1.05 V, respectively.

### 3.2. Photodegradation experiments

#### 3.2.1. Effect of photocatalyst type

**Figure 6:**
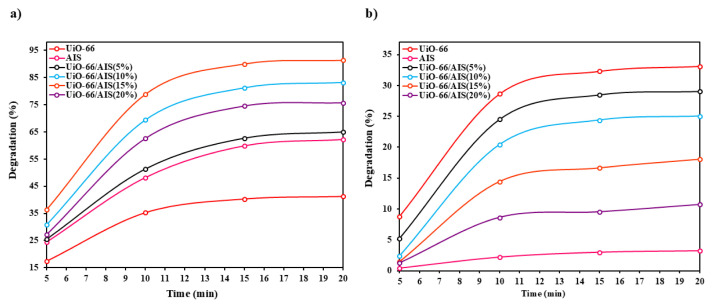
The effects of different photocatalysts in a) degradation and b) adsorption of ENR.

The results of the effect of photocatalyst type in ENR degradation are presented in Figure 6a. The ENR degradation efficiency is the highest for UiO-66/AIS (15%). After 20 min, this value was equal to 91.34%. Increasing the amount of AIS photocatalyst above 15% decreased the absorption capacity at the surface of UiO-66, followed by a reduction in the removal efficiency. By increasing the amount of photocatalyst particles on the surface of UiO-66, the pores on the surface become blocked and the available sites for pollutant adsorption are lost. The adsorption of the synthesized photocatalysts in the dark is shown in Figure 6b. The results show that the AIS photocatalyst had the lowest adsorption of the ENR. The highest adsorption percentage after 15 min was obtained for UiO-66 at 33.11%. Therefore, it can be concluded that the majority of the removal of ENR occurs under visible light irradiation.

#### 3.2.2. Analysis of variance (ANOVA) analysis

**Figure 7:**
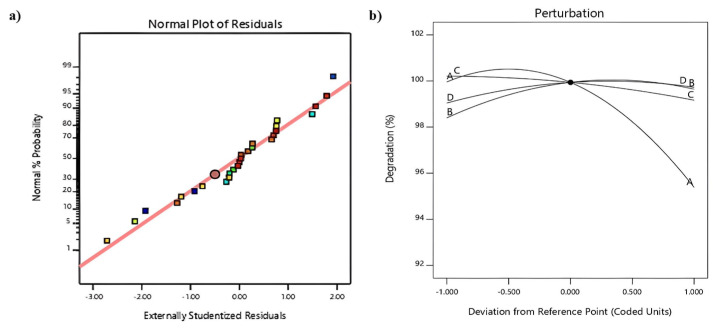
a) Normal probability plot of the studentized residual for degradation of ENR and b) Perturbation plot for degradation ENR.

The combinations of variables in the central composite design (CDD) are shown in Table 4 along with the percentage of ENR degradation for each combination. Moreover, the response of the degradation percentage is expressed as a quadratic function, which is a function of 4 variables, in equation:

The ANOVA results are presented in Table 5. The adjusted R^2^ is 0.9620, and the R^2^ is 0.9825, indicating that the quadratic model provides a good fit. The results in Table 5 show that the predicted model for the response of the ENR degradation percentage test can be used to predict and express the change of the ENR degradation percentage in exchange for different values for the operational parameters. In order to further check the validity of the proposed model, the normal probability graph and perturbation graph for the percentage of ENR degradation are shown in Figure 7a and 7b, respectively.

#### 3.2.3. Effect of pH, photocatalyst mass, ENR concentration, and irradiation time

**Figure 8:**
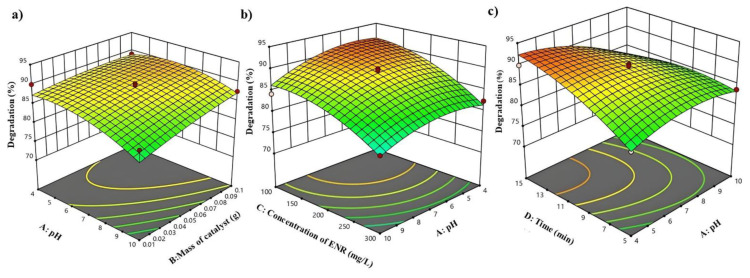
The effect parameters on degradation.

The influence of pH on the elimination of pollutants in wastewater treatment processes is of paramount importance due to its effects on various mechanisms such as adsorption, decomposition, electric charge distribution, and oxidation potential. Moreover, pH plays a critical role in the elimination of antibiotics based on findings from prior studies. It is evident from the data presented in Figure 8 that the optimal degradation efficiency is attained when the pH level is set at 4. This suggests that controlling and maintaining the pH at a specific value is essential for maximizing the removal of contaminants in wastewater treatment systems. The decline in the efficiency of ENR under acidic conditions could potentially be attributed to the rise in hydrogen ions (H^+^) in such environments, which subsequently facilitates the generation of hydroperoxyl (HO_2_) radicals through the interaction with oxygen, ultimately transforming into hydroxyl (OH) radicals. It is worth noting that the acceleration of the degradation process is not solely driven by hydroxyl radicals, but rather by the presence of various free radicals. The emergence of free radical hydrogen (H) within an acidic milieu significantly enhances the overall efficacy of the degradation process, thereby highlighting the crucial role of free radicals in this chemical transformation. Consequently, the intricate interplay between hydrogen ions, free radicals, and radical species underscores the complexity of ENR degradation under low pH conditions. The pH_PZC_ values for the UiO-66/AIS photocatalyst were reported as −27, −21 and −13 eV at different pH values of 5, 7 and 9, respectively. Therefore, it can be concluded that the UiO-66/AIS photocatalyst has better degradation performance at acidic pH due to better electrostatic attraction with ENR.

Initially, the increase in quantity of photocatalyst resulted in a rise ENR degradation percentage; however, as this value continued to increase, the percentage of ENR degradation declined. This can be ascribed to the increase in the turbidity of the solution, the decrease in visible light penetration, the lengthening of the trajectory taken by light photons, and the reduction in the total excitable surface area available for the attachment of the contaminant onto the surface of the photocatalyst. The intricate interplay of these factors explains the fluctuating trend observed in the degradation of ENR with varying amounts of photocatalyst present in the system. The percentage of degradation increases with the increase of ENR concentration until about 200 mg/L, and then decreases. This can be explained by more surface adsorption of ENR molecules at higher concentrations. This reduces the production of hydroxyl radicals and holes and reduces the degradation percentage. As the duration of irradiation is prolonged, the percentage of degradation also increases, but the most significant increase in degradation percentage occurs during the initial 5 min of the processing. Over time, the upward trajectory of ENR degradation diminishes. This phenomenon can be explained by the free radicals generated as a result of the activation of UiO-66/AIS particles and the subsequent generation of hydroxyl free radicals, which are capable of breaking down these compounds, consequently leading to a reduction in the level of ENR degradation. The decline in the degradation rate as time progresses could be due to the depletion of the active radicals or the saturation of the degradation reactions, resulting in a less pronounced impact on the ENR pollutant as the process continues. The results that were deemed optimal indicated that a pH level of 6.39, a mass of photocatalyst measuring 0.015 g, an ENR concentration of 108.52 mg/L, and a duration of 12.13 min, all contributed to the UiO-66/AIS photocatalyst’s capacity to attain a degradation level of 94.32%.

#### 3.2.4. Kinetic study

**Figure 9:**
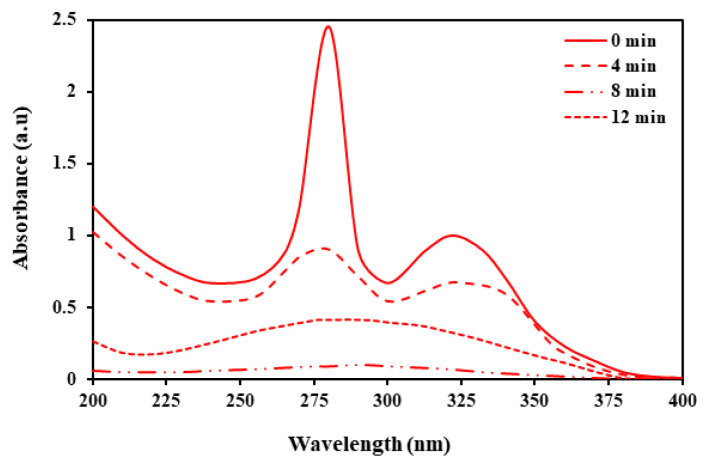
ENR UV-Vis spectra before and after degradation of the photocatalytic process under optimal conditions.

Figure 9 illustrates the outcomes of the ENR UV-Vis spectra analysis, both pre- and postdegradation of the photocatalytic process, in accordance with the optimal conditions. According to the data shown in Table 6, the pseudo-second-order kinetic model displays the highest correlation coefficient among the various models considered. This significant finding indicates a strong relationship between the experimental data and the theoretical model.

#### 3.2.5. Photocatalyst recovery

**Figure 10:**
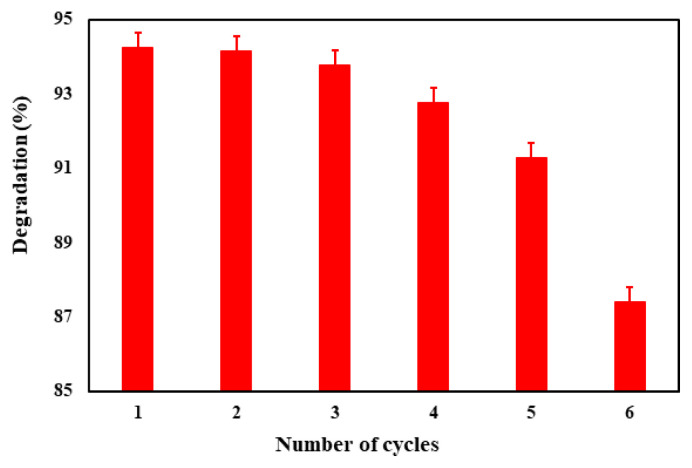
The effect of number of cycles.

One of the crucial factors to consider when utilizing a photocatalyst within the industry pertains to the maintenance of its photocatalytic efficacy following multiple cycles of operation. In order to address this concern, a thorough examination was conducted to assess the recovery and reuse potential of the photocatalyst under optimal operating conditions, spanning a total of 6 operational periods. The findings from the investigation revealed a minimal decrease in degradation efficiency, amounting to a mere 7% reduction after the completion of 6 operational cycles. Moreover, it was observed that even after the 6th cycle, the photocatalytic efficiency remained impressively high, registering above 87% (Figure 10).

#### 3.2.6. Photodegradation pathways of ENR

**Figure 11:**
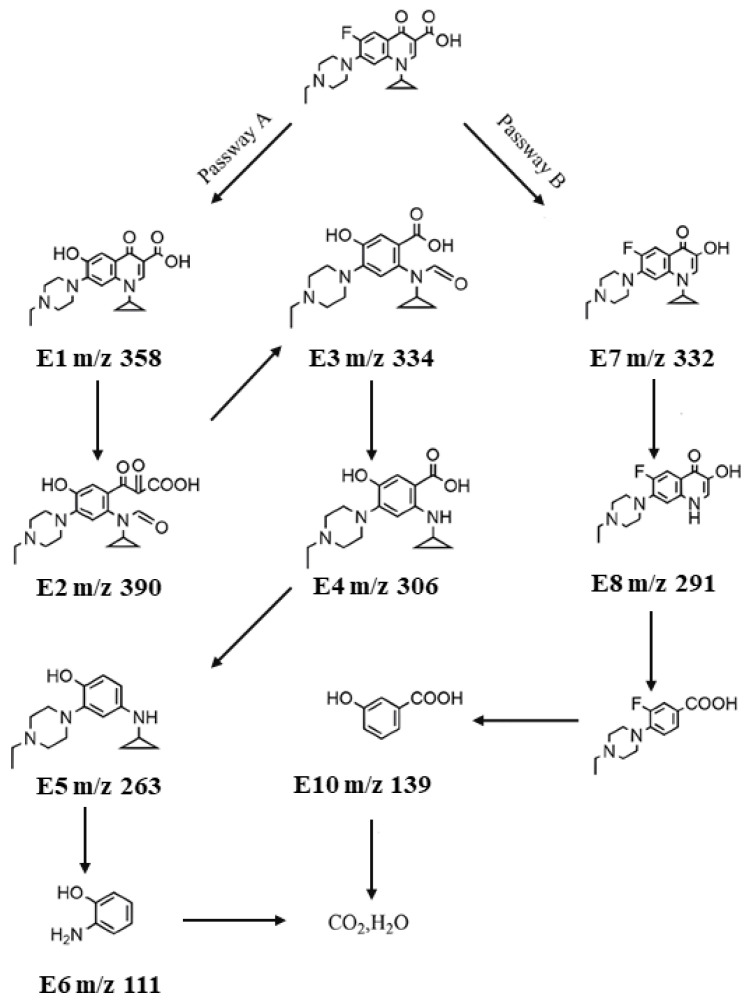
Proposed photodegradation pathways of ENR with UiO-66/AIS p–n junction.

Two degradation mechanisms of ENR are illustrated in Figure 11. ENR molecules with piperazine rings and COO– groups are easily adsorbed and activated on photocatalysts. The decarboxylation process alongside the elimination of the pyridine moiety represents the predominant mechanisms of cleavage in the degradation pathway of ENR. Pathway A involves hydroxyl replacing F– in ENR to form E_1_, followed by cracking of the pyridine ring to generate E_2_. Through a decarboxylation reaction, the production of E_3_, E_4_, and E_5_ occurs, followed by the gradual elimination of both the piperazine and cyclopropyl rings to generate E_6_. Pathway B involves the decarboxylation of ENR to yield E_7_, followed by the removal of the cyclopropyl ring to produce E_8_, and ultimately the cracking of the pyridine and piperazine rings to create E_9_ and E_10_, respectively. These intermediates were then mineralized and transformed into CO_2_, H_2_O, NO_3_−, F, and other minor molecules through further photocatalytic degradation reaction.

The present study compares the photocatalytic activity of UiO-66/AIS with that reported in the literature. The results obtained demonstrate that the UiO-66/AIS p–n junction possesses the capability to degrade almost 94.23% of ENR much more expeditiously than the documented reports in the literature. This degradation phenomenon has been observed to take place within a time frame of 12 min of irradiation. The UiO-66/AIS p–n junction’s exceptional performance can be primarily attributed to its relatively high surface area, which measures approximately 543.14 m^2^ g^−1^ (Table 7) [44–47].

## 4. Conclusion

To assess the degradation rate of the ENR pollutant, a photocatalyst known as UiO-66/AIS (15%) p–n junction was employed under visible light conditions. Findings indicate that the UiO-66/AIS (15%) p–n junction photocatalyst exhibited significant efficacy in breaking down the ENR drug when exposed to visible light. The efficiency of the synthetic photocatalyst utilized in this study is notably high, leading to rapid ENR degradation. Through the utilization of a modest quantity of photocatalyst and exposure to visible light, a considerable level of degradation can be attained. The research examined and fine-tuned the impact of key variables such as pH, photocatalyst quantity, initial pollutant concentration, and irradiation duration on degradation efficiency. RSM was employed to optimize the influential factors on the targeted pollutant degradation efficiency. One of the advantages of the RSM is the optimization of factors affecting the process that, in this study, are a pH of 6.39, 0.015 g of photocatalyst, ENR concentration of 108.52 mg/L, a duration of 12.13 min, and the pollutant concentration 4.80 mg/L. Under these conditions, optical degradation of ENR by UiO-66/AIS (15%) was observed to be 94.23%. Based on the results of the Mott–Schottky examination, it was determined that the values of E_CB_ and E_VB_ for UiO-66 were 1.72 V and 0.64 V, respectively. Similarly, the respective values for AIS were −0.5 V and 1.05 V. The intermediates were mineralized and converted into diverse molecules through photocatalytic degradation. According to the findings, the kinetics of the process align with the pseudo-second-order model, exhibiting a strong correlation coefficient. The results clearly confirm that the kinetics of the process are in line with the pseudo-second-order model that is characterized by a remarkably elevated correlation coefficient. Based on the outcomes of the conducted experimental investigation, we conclude that the UiO-66/AIS p–n junction photocatalyst exhibits a remarkable level of effectiveness and a superior level of performance in a considerably minimized span of time, especially with regard to the elimination of organic pollutants, notably antibiotics.

## Figures and Tables

**Table 1:** Independent variables and their levels in the experimental.

Independent variables	Coded symbols	Levels
pH	X_1_	4 7 10
Mass of catalyst (g)	X_2_	0.01 0.055 0.1
Concentration (mg/L)	X_3_	100 200 300
Time (min)	X_4_	5 10 15

**Table 2:** Synthesized particle size.

Method	UiO-66 (nm)	AIS (nm)	UiO-66/AIS (nm)
Debye–Scherrer	25.51	20.34	18.63
Williamson–Hall	20.03	15.27	36.83
Size-strain plot	16.56	11.50	20.06

**Table 3:** Results of BET-BJH analysis for samples.

Sample	a_S_, BET (m^2^ g^−1^)	Total pore volume (cm^3^ g^−1^)	Mean pore diameter (nm)
UiO-66	792.79	0.509	2.605
AIS	59.21	0.264	1.240
UiO-66/AIS (15%)	543.14	0.376	2.776

**Table 4:** The CCD for the 4 independent variables.

STD	Run	pH	Mass of catalyst (g)	Concentration of ENR (mg/L)	Time (min)	Degradation (%)
6	1	7	0.055	300	5	78.21
19	2	4	0.055	300	10	82.29
3	3	4	0.1	200	10	89.02
21	4	7	0.01	200	5	75.42
5	5	7	0.055	100	5	89.75
17	6	4	0.055	100	10	89.99
26	7	7	0.055	200	10	89.93
22	8	7	0.1	200	5	88.84
15	9	7	0.01	300	10	72.21
27	10	7	0.055	200	10	89.54
12	11	10	0.055	200	15	80.42
18	12	10	0.055	100	10	84.21
4	13	10	0.1	200	10	87.53
25	14	7	0.055	200	10	87.21
13	15	7	0.01	100	10	93.85
1	16	4	0.01	200	10	90.02
2	17	10	0.01	200	10	84.20
9	18	4	0.055	200	5	81.32
10	19	10	0.055	200	5	83.72
24	20	7	0.1	200	15	89.35
14	21	7	0.1	100	10	89.54
23	22	7	0.01	200	15	90.53
20	23	10	0.055	300	10	78.76
7	24	7	0.055	100	15	89.42
8	25	7	0.055	300	15	87.91
16	26	7	0.1	300	10	84.42
11	27	4	0.055	200	15	89.84

**Table 5:** ANOVA for analysis of variance and adequacy of the quadratic model.

Source	Sum of squares	Degree of freedom	Mean square	F-value	p-value Prob >F	
Model	634.04	14	45.29	5.88	0.0020	significant
A- pH	46.57	1	46.57	6.05	0.0301	
B- Mass of catalyst	42.08	1	42.08	5.46	0.0376	
C- Concentration of ENR	233.73	1	233.73	30.34	0.0001	
D- Time	76.05	1	76.05	9.87	0.0085	
AB	4.69	1	4.69	0.6085	0.4505	
AC	1.27	1	1.27	0.1643	0.6924	
AD	34.93	1	34.93	4.53	0.0546	
BC	68.23	1	68.23	8.86	0.0116	
BD	53.29	1	53.29	6.92	0.0220	
CD	25.15	1	25.15	3.26	0.0959	
A^2^	26.53	1	26.53	3.44	0.0882	
B^2^	1.49	1	1.49	0.1939	0.6675	
C^2^	28.84	1	28.84	3.74	0.0769	
D^2^	17.36	1	17.36	2.25	0.1592	
Residual	92.44	12	7.70			
Lack of fit	88.11	10	8.81	4.07	0.2131	not significant
Pure error	4.33	2	2.16			
Corrected total	726.48	26				

**Table 6:** Results of kinetic models.

Sample	Photocatalyst dose (mg)	Concentration of pollutant (mg/L)	Irradiation time (min)	Light source	Pollutant	Efficiency (%)	Ref

TiO_2_ nanosheets	10	10	30	300 W Xe	ENR	98.6	[44]
Cd_0.5_Zn_0.5_S/Bi_2_MoO_6_	10	10	40	300 W Xe	ENR	76.3	[45]
TiO_2_-modified biochar	250	100	60	15 W UV	ENR	85.25	[46]
Ag_2_O/CeO_2_	50	10	30	300 W Xe	ENR	87.11	[47]
UiO-66/AIS	15	108	12	400 W LED	ENR	94.23	Current study

**Table 7:** Comparison between the photocatalytic performance of UiO-66/AIS p–n junction and other relevant researches.

Kinetic model	Equation			
		*k* *_1_*	*q* *_e_*	R^2^
Pseudo-first-order model	$\text{ln}(q_{e}-q_{t})=lnq_{e}-k_{1}t$	0.321 *k_2_*	40.54 *q_e_*	0.8786 R^2^
Pseudo-second-order model	$\frac{t}{q_{t}}=\frac{1}{k_{2}q_{e}^{2}}+\frac{t}{q_{e}}$	0.018 *k_i_*	85.55 *C*	0.9941 R^2^
Intraparticle diffusion control	$q_{t}=k_{i}t^{1/2}+C$	6.43	58.40	0.9936
